# Machine Learning in Prediction of Second Primary Cancer and Recurrence in Colorectal Cancer

**DOI:** 10.7150/ijms.37134

**Published:** 2020-01-15

**Authors:** Wen-Chien Ting, Yen-Chiao Angel Lu, Wei-Chi Ho, Chalong Cheewakriangkrai, Horng-Rong Chang, Chia-Ling Lin

**Affiliations:** 1 Division of Colorectal Surgery, Department of Surgery, Chung Shan Medical University Hospital, Taiwan; 2 Institute of Medicine, Chung Shan Medical University, Taiwan; 3 School of Nursing, Chung-Shan Medical University, Taiwan; 4 Department of Gastroenterology, Jen-Ai Hospital, Taichung, Taiwan; 5 Division of Gynecologic Oncology, Department of Obstetrics and Gynecology, Faculty of Medicine, Chiang Mai University, Thailand; 6 Division of Nephrology, Department of Internal medicine, Chung Shan Medical University Hospital, Taiwan; 7 School of Medicine, Chung Shan Medical University; 8 Department of Nutrition, Jen-Ai hospital, Taichung, Taiwan

**Keywords:** colorectal cancer, second primary malignancy, machine learning

## Abstract

Background: Colorectal cancer (CRC) is the third commonly diagnosed cancer worldwide. Recurrence of CRC (Re) and onset of a second primary malignancy (SPM) are important indicators in treating CRC, but it is often difficult to predict the onset of a SPM. Therefore, we used mechanical learning to identify risk factors that affect Re and SPM.

Patient and Methods: CRC patients with cancer registry database at three medical centers were identified. All patients were classified based on Re or no recurrence (NRe) as well as SPM or no SPM (NSPM). Two classifiers, namely A Library for Support Vector Machines (LIBSVM) and Reduced Error Pruning Tree (REPTree), were applied to analyze the relationship between clinical features and Re and/or SPM category by constructing optimized models.

Results: When Re and SPM were evaluated separately, the accuracy of LIBSVM was 0.878 and that of REPTree was 0.622. When Re and SPM were evaluated in combination, the precision of models for SPM+Re, NSPM+Re, SPM+NRe, and NSPM+NRe was 0.878, 0.662, 0.774, and 0.778, respectively.

Conclusions: Machine learning can be used to rank factors affecting tumor Re and SPM. In clinical practice, routine checkups are necessary to ensure early detection of new tumors. The success of prediction and early detection may be enhanced in the future by applying “big data” analysis methods such as machine learning.

## Introduction

Colorectal cancer (CRC) is the third most common cancer in the world and the fourth most common cause of death overall 1. In Taiwan, CRC is the most common type of cancer, with crude incidence rate approximately 46.7 in colon cancer and 27.9 in rectal cancer per 100,000 populations 2. The recurrence rate for late-stage CRC is relatively high, but the 5-year relative survival rate varies from 88.1% (stage I) to 65.8% (stage III) 3 worldwide.

Recurrence and second primary malignancies (SPMs) affect the survival of CRC patients. Numerous studies have documented increases in the incidence and recurrence of CRC as well as increases in second primary cancers, including lung, head, neck, and gastric cancer 4-6. It has been suggested that second primary cancers may affect the recurrence of the primary cancer 7; however, no conclusive data to this effect have been reported in CRC.

Early detection of tumor recurrence and SPMs is essential to improving health outcomes of cancer patients. To detect SPMs, valid and reliable prediction tools are necessary. Traditional statistical methods, such as the chi-square test, multiple linear regression test, ANOVA, and *t*-test, have been used, but this requires a rigorous research design and clear, explicit hypotheses. In addition, the calculations cannot be modified when parameters change without redesign.

In recent years, machine learning (ML) has emerged as an alternative to express parameters in disease treatment and outcome. ML is a process where an acceptable generalization is obtained by searching through an *n*-*dimensional* space for a given set of biological samples using different techniques and algorithms 8. It has been applied extensively in biomedical research. The two main common types of ML methods are supervised learning and unsupervised learning. Supervised learning can be thought as a classification process, meaning the learning process categorizes the data into a finite set of classes. The expansion of computational tools that allow ML processes has been a key development in the analysis of histological data for CRC 9, 10. In this approach, a computer is first 'trained' using a clinical CRC data set classified by a physician. The data include recurrent and non-recurrent cancer with related clinical factors. The ML method then uses this classification information to develop its own pattern-recognition criteria to identify recurrent tumors. Our research aims to analyze these clinical data using ML models to identify the recurrence of colorectal tumors as well as the occurrence of second primary cancers.

## Methods

### Dataset preparation

Our study used data accessed from the Cancer Registry in three medical centers. Individuals with ICD-09 codes 153~154 who were diagnosed with CRC between 2004 and 2012 and were 18 years or older at the time of diagnosis were selected for inclusion in the study. We used the cancer sequence number and the recurrence status of primary CRC as the target classification. Patients with CRC alone were selected first and identified using sequence number 1. Patients with an SPM in addition to primary CRC were identified using sequence number 2 and included in the analysis. All CRC patients were classified between 2004 and 2012 and the study is conducted as a time to event analysis.

**Figure 1:**
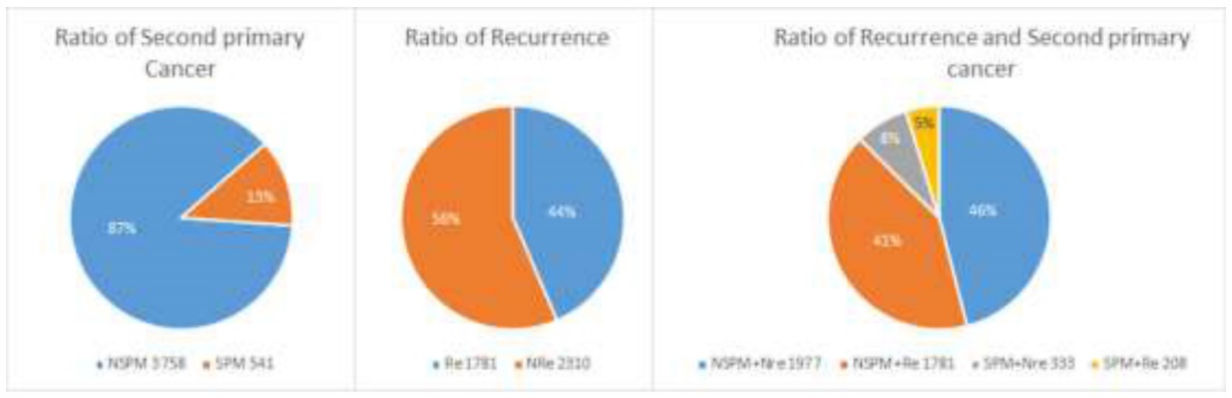
Second primary malignancies and recurrence of colorectal cancer (CRC) in the study population.

A total of 4299 patients with primary CRC were enrolled. As shown in Figure 1, 541 patients had at least one SPM (“SPM” group) and 3758 had no SPM (“NSPM”). In addition, 1989 patients had recurrent CRC (“Re” group) and 2310 had no recurrence (“NRe”). To evaluate both parameters, the total sample was divided into four groups (Figure 1): SPM+Re (208), NSPM+Re (1781), SPM+NRe (333), and NSPM+NRe (1977).

### Features

Risk factors previously reported to be associated with CRC tumor recurrence and SPM include tumor size, morphology, differentiation 11, previous radiation therapy 12, and smoking 13. Because a ranking of these factors was not found in the reported studies, we tried to rank risk factors based on data from the cancer registry using machine learning. We used correlation analysis to examine the following 20 features (risk factors): patient age, primary site, histology, behavior code, differentiation, tumor size, pathologic stage (pStage), surgical margins, surgical procedure, radiation therapy, pre-operative radiation therapy, regional body order, highest and lowest dose of radiotherapy, maximum and minimum times of radiotherapy, body mass index (BMI), smoking, areca consumption, and drinking. Those factors not only be the risk factor of CRC but also have important role in other cancer. Such as drinking, betel used, previous radiation therapy have contribution in hepatocellular cancer, oral cancer and lung cancer. We analyzed the relationships between these features and recurrence of CRC and/or occurrence of SPM.

### Classifier

Support Vector Machines (SVMs) are supervised learning algorithms that can be used in binary classification problems and have been applied in many fields. SVMs maps feature vectors to a high-dimensional feature space, which classifies samples by searching for an optimal hyperplane and can divide the samples into different spatial areas. We uses LIBSVM (A Library for Support Vector Machines)^14^ with the radial basis function kernel to construct predictive models and optimize the C and γ parameters of each model.

Reduced Error Pruning Tree (REPTree) uses regression tree logic and creates multiple trees in different iterations. After this process, the optimal (or representative) decision from all generated trees were identified. The mean squared error of the prediction was used to prune the tree. REPTree offers a fast decision tree learning method and builds a decision/regression tree based on information gain or by minimizing the variance. The information gain was used as the splitting criterion and the reduce-error pruning method was also applied to reduce the size of the decision trees. Values were sorted once for numeric attributes, and then, to improve accuracy, all subtrees were visited in a bottom-up manner until no subtrees were replaced with leaves.

### Feature selection

We applied the feature selection tool developed by the LIBSVM (A Library for Support Vector Machines)^14^ team to determine the discrimination of single vectors in different categories by the scoring of the F-test (F-score), and then ranked the significance of vectors by the F-score 15. Given training vectors *xk*, where *k* = 1,…,*m*, if the number of positive and negative instances are *n*+ and *n*-, respectively, the F-score of the *i*th feature is defined as:


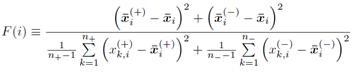
 (1)

where 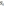, 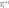, 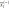
are the average of the *i*th feature of the whole, positive, and negative data sets, respectively; 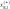 is the *i*th feature of the *k*th positive instance; and 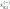
is the *i*th feature of the *k*th negative instance. The larger the F-score, the more likely this feature is discriminative. This score was used as a feature selection criterion.

### System workflow

**Figure 2:**
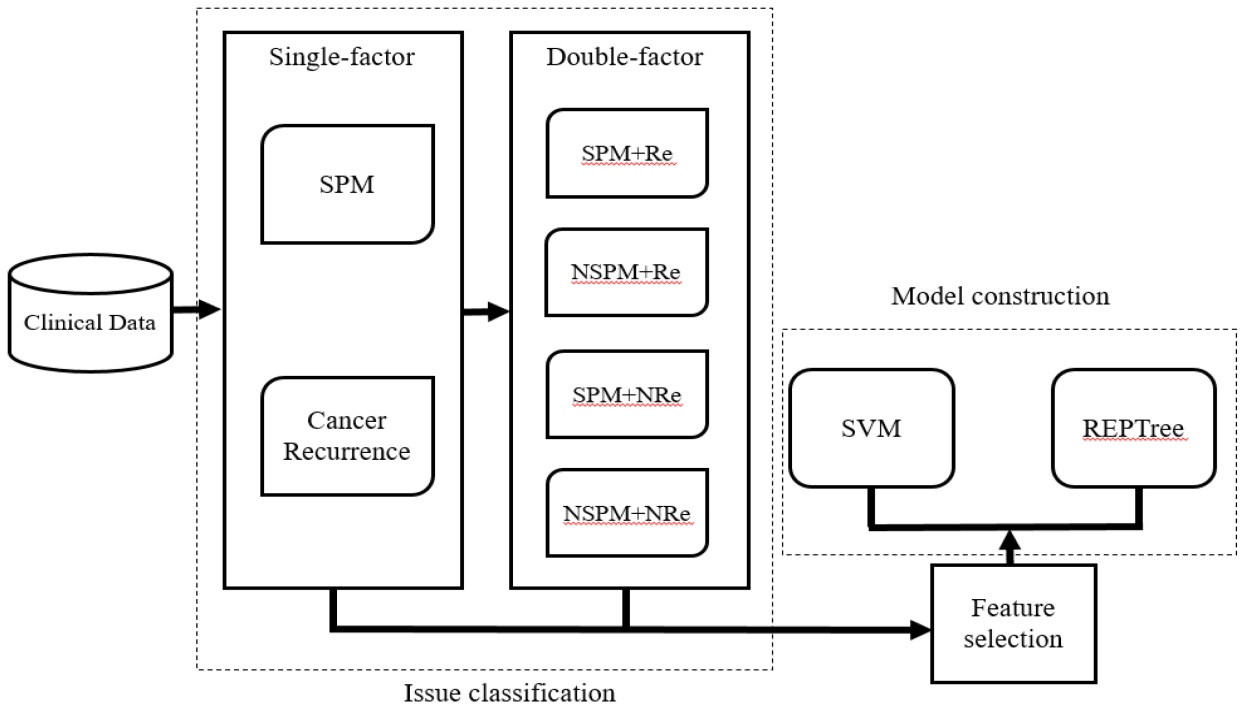
Workflow for model construction from clinical data.

After selecting patients for inclusion and dividing them into the four classification groups, the significant ranking of the 20 features was analyzed using the LIBSVM feature selection mode of the training dataset. Then, based on the performance of the predictive model, we selected candidate features that could assist in classification of recurrence and SPM. The LIBSVM algorithm was used as the initial classifier for model construction. Although it is easy to achieve the best accuracy using LIBSVM, some SVM algorithms are complicated and difficult to understand. Therefore, Reduced Error Pruning Tree (REPTree) was applied to provide possible rules for auxiliary analysis. In the study, we choose 16. In our initial analysis, we constructed separate models assessing the relationship between the 20 features and either SPM or recurrence of CRC. In the subsequence analysis, SPM and Re were analyzed together and four models (SPM+Re, NSPM+Re, SPM+NRe and NSPM+NRe) were built (Figure 2). We implemented the REPTree and SVM algorithms using WEKA 17, 18 and LIBSVM, and employed a 10-fold cross-validation to evaluate model performance.

### Evaluation

The predictive ability of each system was evaluated for accuracy (Acc), sensitivity (Sn), specificity (Sp), and the Matthews correlation coefficient (MCC), which were defined as follows:


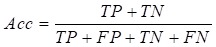
 (2)


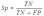
 (3)


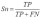
 (4)


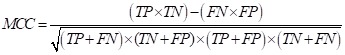
 (5)

Where TP, FP, FN, and TN represent true and false positives and negatives. Acc evaluates the prediction accuracy of positive and negative data, whereas Sn and Sp evaluate the accuracy of the prediction of positive and negative data, respectively. The values of these parameters range from 0 to 1. MCC is suitable for assessing the dataset for imbalance between positive and negative data and ranges from -1 to 1. The model performance is better when MCC is closer 1 and vice versa.

## Results

### Significant features by Re

**Figure 3:**
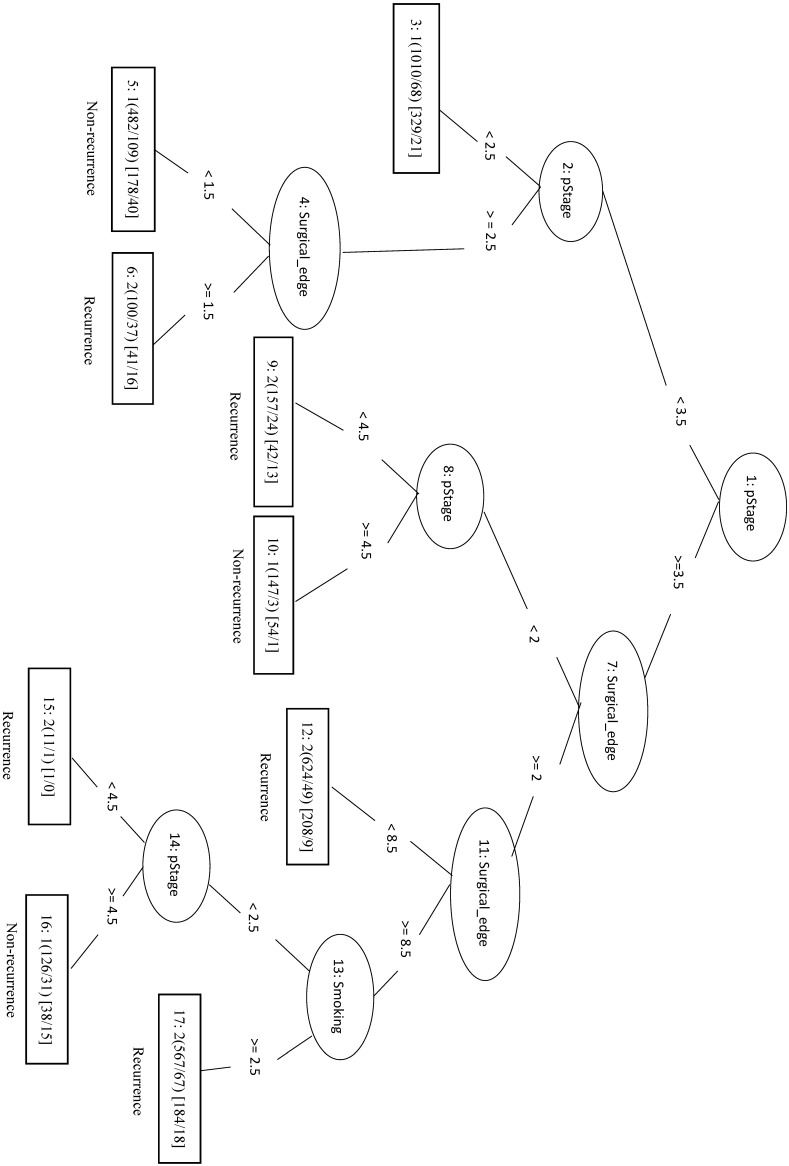
Decision tree of important factors for recurrent CRC classification using the REPTree_3F model.

In this study, we identified 1989 out of 4299 total cases that had recurrent CRC (the Re group) and 2310 cases with no recurrence (the NRe group). Model evaluation data are shown in Table 1. The MCC of the initial LIBSVM model including all 20 features was 0.709. When only the top four features (pStage, surgical margin, smoking, and drinking) were included in the model (LIBSVM_4F), the MCC improved from 0.709 to 0.753 and the Acc improved from 0.856 to 0.877. Removing drinking from the analysis (LIBSVM_3F) increased the MCC, Sn, Sp, and Acc to 0.755, 0.891, 0.863, and 0.878, respectively. A similar increase was observed when compared REPTree classifier models involving either all 20 features or the same three selected features (pStage, surgical margin, and smoking). Using only the top three features (REPTree_3F), the Acc increased from 0.875 to 0.878 and the MCC increased from 0.748 to 0.754. The REPTree_3F model is shown in Figure 3.

### Significant features by SPM

In addition, we identified 541 cases with SPM and 3758 cases without SPM (NSPM). Because using two datasets with drastically different sizes (SPM/NSPM ratio = 1:6.95) can affect data quality and hinder models training process, we constructed models using three different ratios (1:1, 1:1.5, and 1:6.95) and compared their accuracy. Our results are shown in Table 2. Using the model at the 1:6.95 ratio, LIBSVM improved the accuracy compared to the REPTree classifier by adjusting the unbalanced parameters (LIBSVM - w1 1 - w2 10) and the Acc was 0.635 (Table 2).

To generate models using ratios of 1:1, the SPM patients were combined with 541 randomly selected patients from NSPM to form training dataset. Under the condition, the MCC of the LIBSVM model was 0.324 and the Sp and Sn were both greater than 0.6. The LIBSVM_F model, employing only the top eight features (behavior code, differentiation, regional body order, patient age, areca, surgery, radiation therapy, and lowest dose), had the equivalent performance outcome as the model using all 20 features.

**Figure 4:**
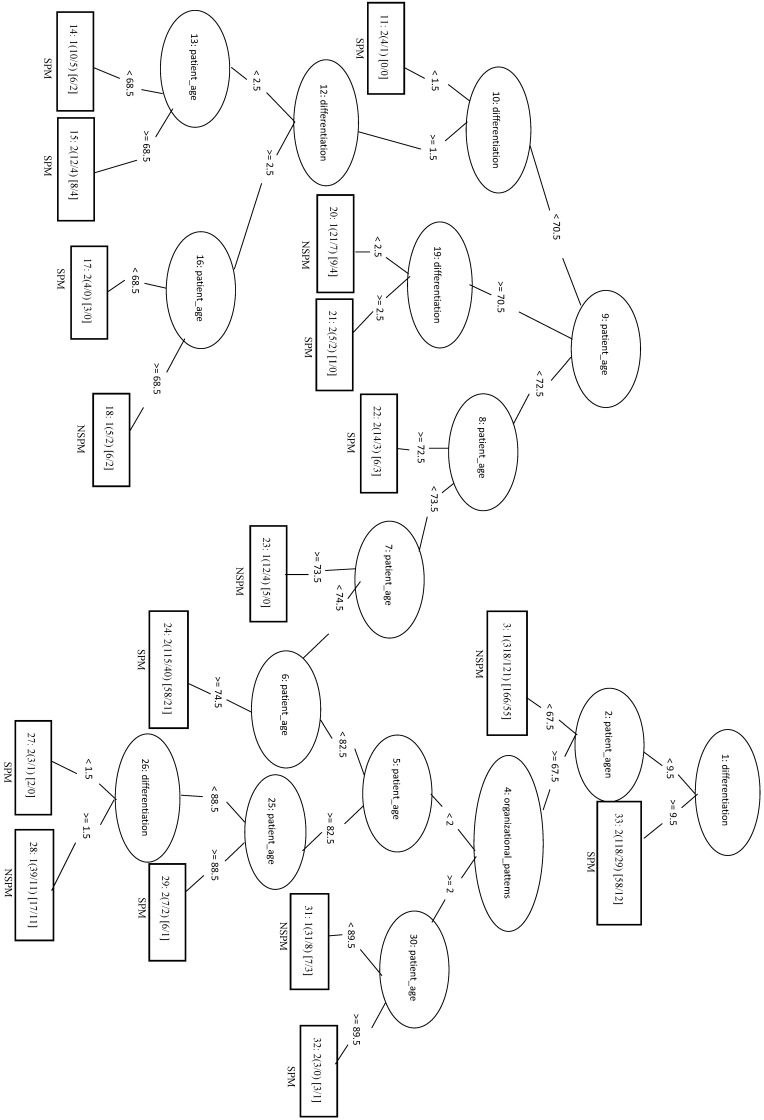
Decision tree of important features for SPM classification using the REPTree-OP model.

Applying the REPTree algorithm with the 1:1 ratio gave an MCC of 0.282. When an optimized model (REPTree_OP) was constructed using only the top three features (patient age, differentiation and organizational patterns), the MCC increased to 0.294 and the Sn increased to 0.706. In the REPTree_OP decision tree in Figure 4, differentiation values > 9.5 were classified as SPM, whereas differentiation values < 9.5 lead to leaf nodes of organizational patterns and patient age for classifying SPM versus NSPM.

### Significant factors by Re and SPM

Lastly, we considered the two results of recurrent CRC and SPM together using the four conditions SPM+Re, SPM+NRe, NSPM+Re, and NSPM+NRe. Data are presented in Table 3.

### Second Primary Malignancy +Recurrence

**Figure 5:**
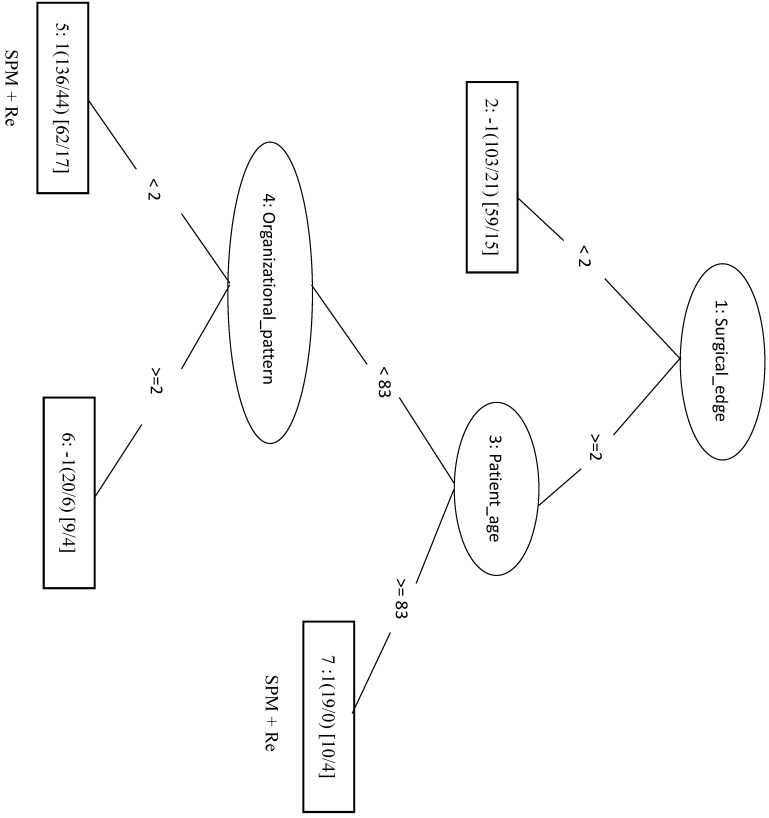
Decision tree of important factors for SPM + Re classification using the REP Tree_F model.

We randomly selected 70 samples from each of the other three classes (total of 210 negative samples) and combined these with the 208 SPM+Re (positive) samples into the training dataset. When only the top four factors (surgical margins, pStage, areca, and drinking) were included in the analysis (LIBSVM_F), the MCC was 0.466 and the Acc was 0.732. In the REPTree model, the MCC, Acc, and Sn were 0.448, 0.722, and 0.774, respectively. This was the same as the SVM model applying only the three features of surgical margins, organizational patterns, and patient age. The decision tree shows that rules of surgical margins ≥2, patient age <83, and organizational patterns <2 *or* surgical margins ≥2 and patient age ≥83 can be classified into SPM+Re (Figure 5).

### No Second Primary Malignancy +Recurrence

**Figure 6:**
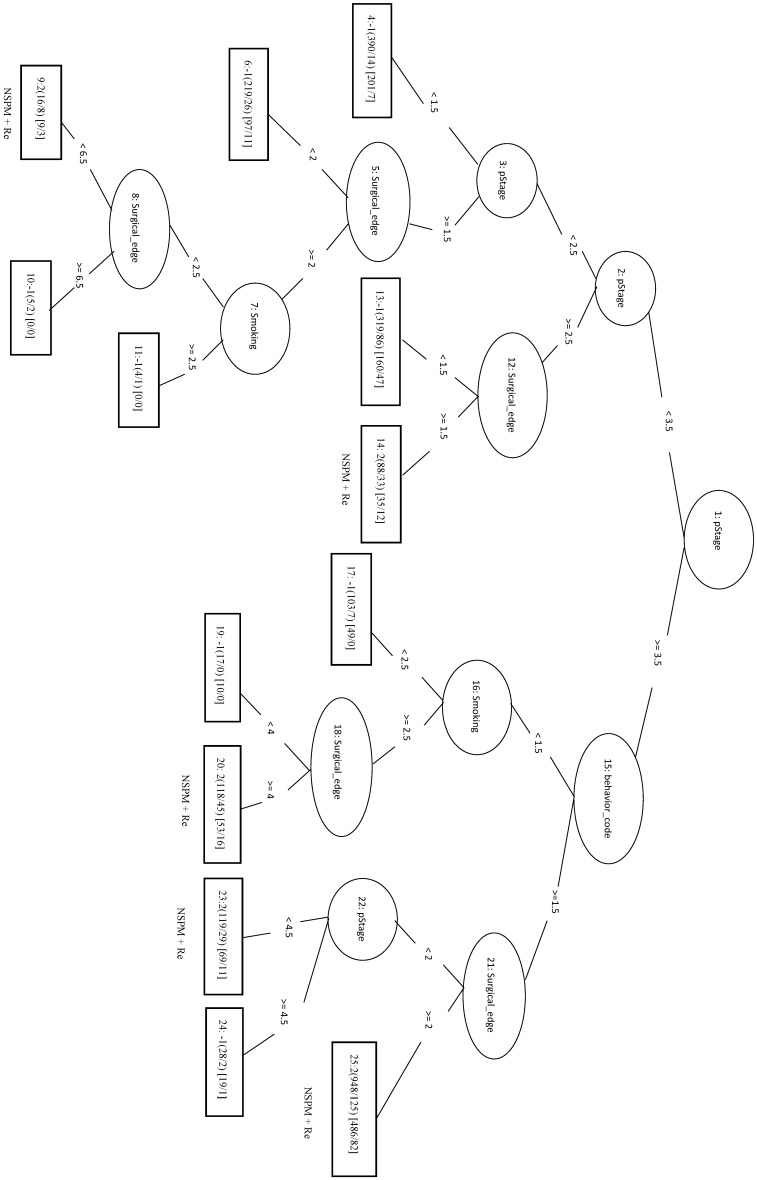
Decision tree of important factors for NSPM+Re classification using the REPTree_F model.

We randomly selected a total of 1781 samples from three remaining classes as negative data and combined them with the 1781 NSPM+Re (positive) samples into the training dataset. When four major factors (pStage, surgical margins, behavior code, and smoking) were selected for analysis, the MCC of the LIBSVM_F model was 0.676 and the Acc was 0.836. Using the same four factors in the REPTree_F model gave similar values for MCC and Acc. Both models also achieved a sensitivity above 0.88. The rules of the decision tree for REPTree_F are shown in Figure 6.

### Second Primary Malignancy + No Recurrence

**Figure 7:**
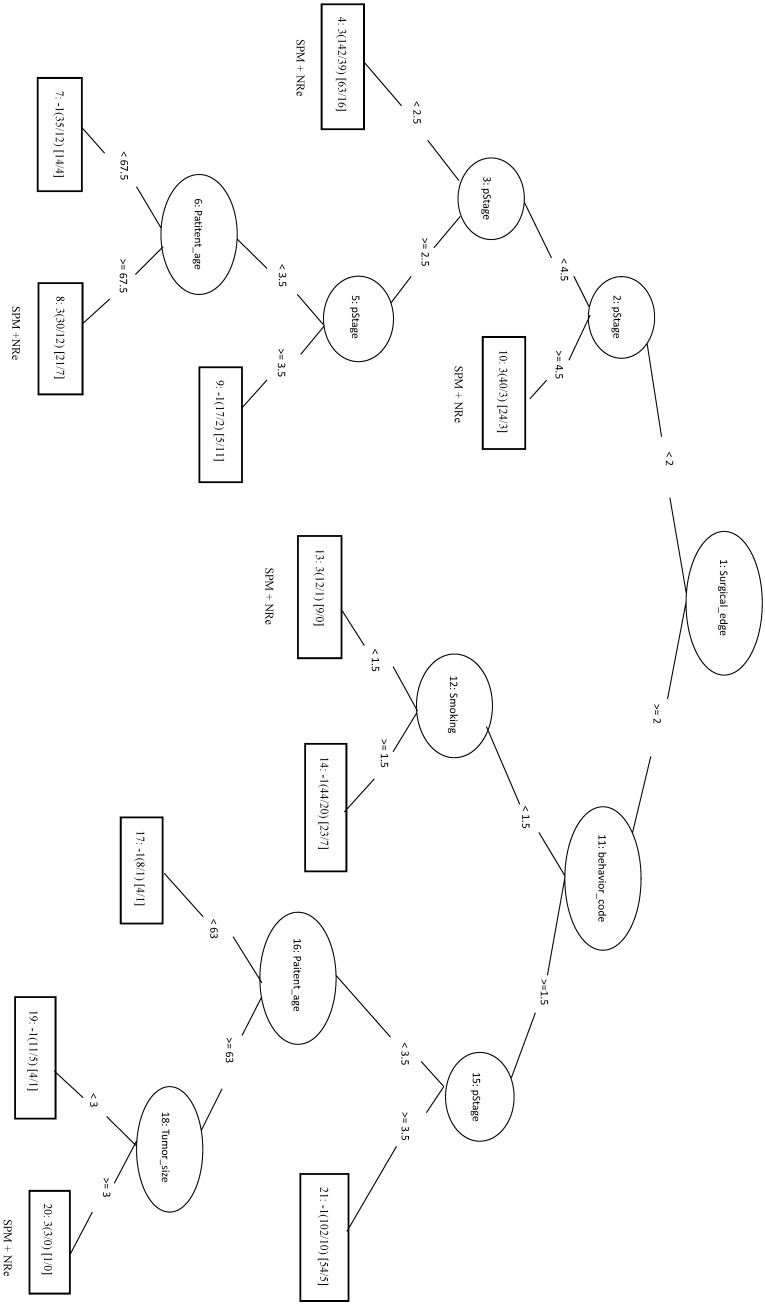
Decision tree of important factors for SPM+NRe classification using the REPTree_OP model.

The 333 samples from SPM+NRe class (positive data) and 111 samples randomly selected from each the other three classes (negative data) were combined into the training dataset. In the LIBSVM_F model using the top three features (behavior code, pStage, and surgical margins), the MCC was 0.494 and the Sn was 0.679. For the REPTree model, the MCC was 0.446 and the Sn was 0.757. When surgical margins, pStage, tumor size, behavior code, patient age, and smoking are used in the REPTree model after parameter optimization (REPTree_OP), the MCC was 0.504 and the Sn was 0.823 (increases of 0.058 and 0.066, respectively). Compared to the SVM model with three features, the REPTree_OP model was similar in MCC and had a higher Sn (by 0.144), but had a lower Sp (by 0.135). The rules of the decision tree for REPTree_OP are shown in Figure 7.

### No Second Primary Malignancy + No Recurrence

**Figure 8:**
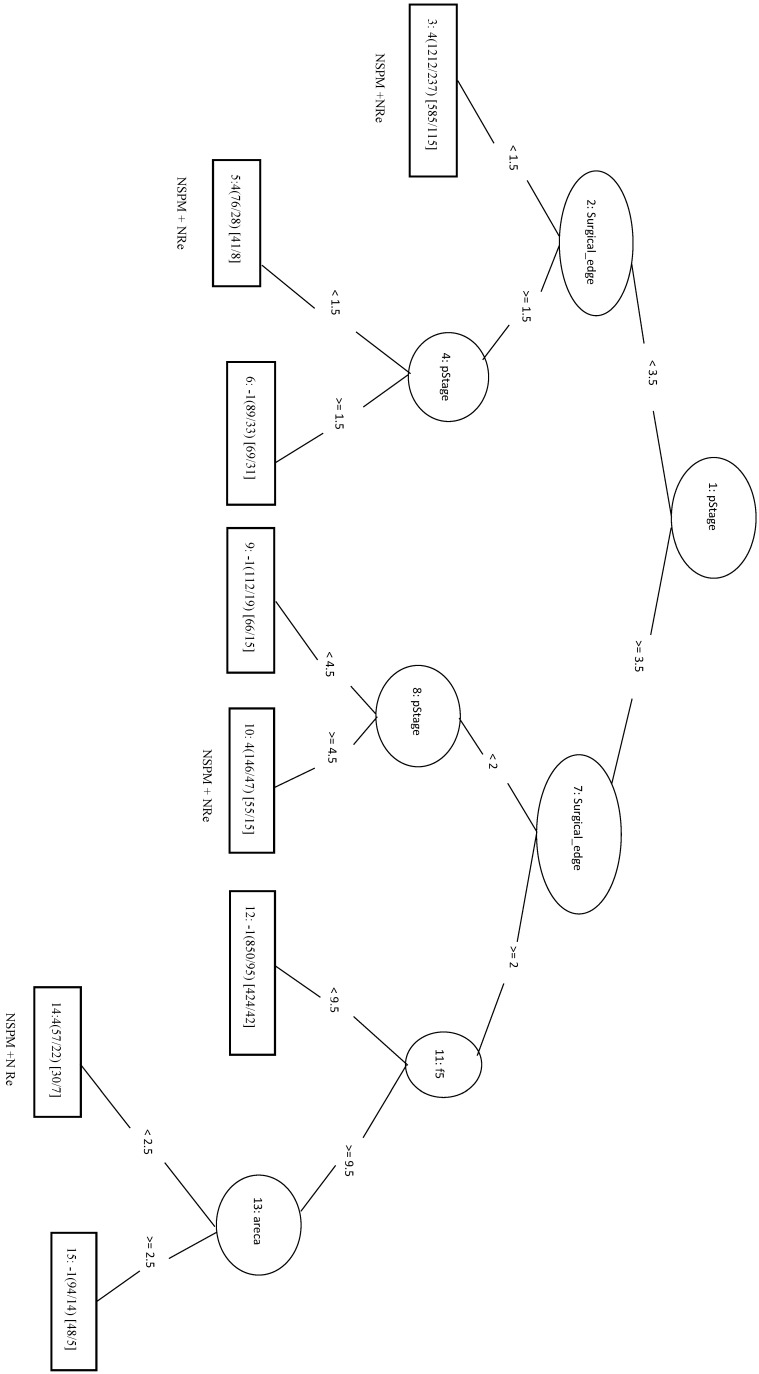
Decision tree of important factors for NSPM+NRe classification using the REPTree_OP model.

The 1977 samples (positive data) and were combined with 1977 randomly selected samples from other three classes as negative data for the training data. The top four features were pStage, surgical margins, differentiation, and tumor size. The LIBSVM model using all features had an MCC of 0.592. Using only pStage and surgical margins in the model improved performance of MCC (0.613), Sn (0.862), and Sp (0.746). In REPTree, the MCC increased from 0.612 before parameter optimization to 0.630 after parameter optimization using pStage, surgical margins, differentiation, and areca. The performance of REPTree was better than that of SVM. The rules of the decision tree for REPTree_OP are shown in Figure 8.

## Discussion

Our study explored risk factors for predicting CRC recurrence and SPM and discovered that the four most important factors were pStage, surgical margins, smoking, and drinking. However, sensitivity (Sn) decreased slightly when drinking was removed from the analysis and decreased further when smoking was removed. These findings suggest that both drinking and smoking have an effect on recurrence and SPM. On the other hand, both surgical margins and pStage were significant factors using both SVM and REPTree models for classification.

In classification for SPM, when eight features (behavior code, differentiation, regional body order, patient age, areca, surgery, radiation therapy, and lowest dose) were selected to construct the REPTree model, the MCC (0.229, data not shown) outperformed the REPTree model without feature selection (MCC = 0.282, Table 2). In addition, the REPTree algorithm with parameter optimization used only three features (patient age, organizational patterns, and differentiation) to improve performance. Both methods used patient age and differentiation as factors in constructing the models. When only these two factors were used, the accuracy was relatively high for predicting SPM (0.708), but dropped to 0.518 for predicting NSPM. These findings suggested that other factors may be involved in classifying NSPM.

For all four groups in the combined analysis of recurrence and SPM, pathologic stage and surgical margins were included in all models. This highlights the clinical importance of these two factors. On the other hand, primary site was the last feature to be included in all models, indicating that it has little clinical or reference value (Table 4).

Although our results show that selected features can be weighted using mechanical learning to find predicting factors for recurrence and SPM, there were some limitations to the study. Some site-specific factors, such as tumor markers and tumor regression grade, were not included as risk factors. Including these and other factors in machine learning programs may improve the prediction and early detection of recurrence and SPM.

There are some limitations in the study. Risk factors such as family history of breast cancer, Hereditary Nonpolyposis Colorectal Cancer cannot be available. This may affect the result of SPM. The will be enrolled in further study.

## Conclusion

With mechanical learning programs, we have developed a feasible and a robust method to identify factors that are important for predicting recurrence of colorectal cancer and SPM. The four most important factors are pStage, surgical margin, smoking, and drinking. Mechanical learning can be used as an effective medical decision-making tool to improve prognostic and diagnostic accuracy in clinical settings. We strongly recommend that clinicians consider using mechanical learning in diagnosing and treating cancer patients to provide high-quality care.

## Figures and Tables

**Table 1:** Model evaluation for CRC recurrence alone.

Classifier	TP	FP	TN	FN	Sn	Sp	Acc	MCC
LIBSVM	2040	351	1638	270	0.883	0.824	0.856	0.709
LIBSVM_4F	2063	281	1708	247	0.893	0.859	0.877	0.753
LIBSVM_3F	2059	273	1716	251	0.891	0.863	0.878	0.755
REPTree	2035	263	1726	275	0.881	0.868	0.875	0.748
REPTree_3F	2070	286	1703	240	0.896	0.856	0.878	0.754

LIBSVM_3F, REPTree_3F, and LIBSVM_4F models were constructed using feature selection with the top three or four features, respectively.

**Table 2:** Model evaluation for SPM alone.

Ratio	Classifier	TP	FP	TN	FN	Sn	Sp	Acc	MCC
1:6.95	LIBSVM	315	1345	2413	226	0.582	0.642	0.635	0.153
1:1.5	LIBSVM	173	89	723	368	0.320	0.890	0.662	0.261
1:1	LIBSVM	350	175	366	191	0.647	0.677	0.662	0.324
1:1	LIBSVM_F	363	188	353	178	0.671	0.652	0.662	0.324
1:1	REPTree	373	221	320	168	0.689	0.591	0.640	0.282
1:1	REPTree_OP	382	224	317	159	0.706	0.586	0.646	0.294
1:1	REPTree_F	363	240	301	178	0.671	0.556	0.614	0.229

LIMSVM_F and REPTree_F models were constructed using feature selection with the top eight features. REPTree_OP model was constructed [using parameter optimization OR as an optimized model] with the top three features.

**Table 3:** The model evaluation for second primary malignancies and recurrent cancer co-discussion.

	Classifier	TP	FP	TN	FN	Sn	Sp	Acc	MCC
SPM+Re	REPTree	161	69	141	47	0.774	0.671	0.722	0.448
	REPTree_F	169	83	127	39	0.813	0.605	0.708	0.426
	LIBSVM	145	63	148	62	0.700	0.701	0.701	0.402
	LIBSVM_F	161	65	145	47	0.774	0.690	0.732	0.466
NSPM+Re	REPTree	1546	365	1416	235	0.868	0.795	0.832	0.665
	REPTree_F	1572	374	1407	209	0.883	0.790	0.836	0.676
	LIBSVM	1477	365	1416	304	0.829	0.795	0.812	0.625
	LIBSVM_F	1569	370	1411	212	0.881	0.792	0.837	0.676
SMP+NRe	REPTree	252	104	229	81	0.757	0.688	0.722	0.446
	REPTree_OP	274	108	225	59	0.823	0.676	0.749	0.504
	REPTree_F	236	98	235	97	0.709	0.706	0.707	0.414
	LIBSVM	235	99	234	98	0.706	0.703	0.704	0.408
	LIBSVM_F	226	63	270	107	0.679	0.811	0.745	0.494
NSPM+NRe	REPTree	1705	504	1473	272	0.862	0.745	0.804	0.612
	REPTree_OP	1739	505	1472	238	0.880	0.745	0.812	0.630
	REPTree_F	1670	465	1512	307	0.845	0.765	0.805	0.611
	LIBSVM	1700	539	1438	277	0.860	0.727	0.794	0.592
	LIBSVM_2F	1705	502	1475	272	0.862	0.746	0.804	0.613

LIBSVM_F indicates SVM model building with feature selection. REPTree_F indicates REPTree model building with feature selection. REPTree_OP indicates REPTree model building by parameters optimization.

**Table 4:** Order of top ten features by F-score for feature selection

Re	SPM	SPM+Re	NSPM+Re	SPM+NRe	NSPM+NRe
pStage	behavior code	Surgical edge	pStage	behavior code	pStage
Surgical edge	differentiation	pStage	surgical edge	pStage	surgical edge
Smoking	regional body order	areca	behavior code	surgical edge	differentiation
drink	age	drink	smoking	highest dose	tumor size
radiation therapy	areca	Smoking	radiation therapy	radiation therapy	smoking
areca	surgery	BMI	drink	age	drink
differentiation	radiation therapy	age	surgery	lower number of times	radiation therapy
surgery	lowest dose	differentiation	areca	smoking	areca
BMI	organizational patterns	lowest dose	BMI	radiation therapy before surgery	BMI
behavior code	highest dose	tumor size	differentiation	tumor size	surgery
